# An integrated method for color correction based on color constancy for early mural images in Mogao Grottoes

**DOI:** 10.3389/fnins.2022.1024599

**Published:** 2022-12-15

**Authors:** Zhen Liu, Yi-Xuan Liu, Gui-Ai Gao, Yong Kong, Bing Wu, Jin-Xing Liang

**Affiliations:** ^1^School of Communication, Qufu Normal University, Rizhao, Shandong, China; ^2^School of Statistics, Qufu Normal University, Rizhao, Shandong, China; ^3^School of Computer Science and Artificial Intelligence, Wuhan Textile University, Wuhan, China

**Keywords:** color constancy, illumination non-uniformity, color cast correction, Dunhuang murals, homomorphic filter

## Abstract

Restoring the correct or realistic color of a cultural heritage object is a crucial problem for imaging techniques. Digital images often have undesired color casts due to adverse effects caused by unstable illuminant conditions, vignetting, and color changes due to camera settings. In this work, we present an improved color correction method for color cast images that makes the color appear more realistic. It is based on a computational model of the human visual system that perceives objects by color constancy theory; it realizes illumination non-uniformity compensation and chromaticity correction for color cast images by taking into account the color stability of some pigments. This approach has been used to correct the color in Cave 465 of the Mogao Grottoes. The experimental results demonstrate that the proposed method is able to “adaptively correct” color cast images with widely varying lighting conditions and improve the consistency efficaciously. It can achieve improved consistency in the mean CIEDE2000 color difference compared with the images before correction. This colorimetric correction methodology is sufficiently accurate in color correction implementation for cast images of murals captured in the early years.

## Introduction

The human visual perception system has the ability to match objects’ colors in scenes taken under ambient lighting conditions; the color appears to be approximately constant to human observers. A scientific theory can be defined as color constancy; some explanation of color constancy has been confirmed by observation or experiment (Barbur et al., 2004; Foster, 2011; McCann et al., 2014; Gao et al., 2015, 2019). It is part of a larger system of subjective constancy, which is used by the brain to help people perceive objects in changing situations. This ensures that we can recognize objects, which assists in comprehending the world and is important for safety.

Color-accurate image archives of cultural heritage items have played a vital role in their preservation, and scientific studies have been carried out during restoration and renovation. Color constancy is a complex problem for cultural heritage digitization because the image color depends on the spectral reflectance function of the object and the spectral distribution function of the incident light, both of which are generally unknown. Therefore, color constancy became the problem of removing the color of the light that illuminated a scene (Campagnolo and Celes, 2019; Akazawa et al., 2021; Huang et al., 2021). Precise digitization facilitates the preservation of a real object’s color and appearance in optimal circumstances and provides a digital simulation available for research at large (Li et al., 2000, 2013; Granzier et al., 2009; Akazawa et al., 2021). In the early years, conservators and conservation scientists preserved murals by taking pictures using film-based photography because good digital cameras did not have sufficient image quality (Verhoeven, 2016). After that, high-resolution scanning and processing of the negative analog film frames needed to be performed to achieve digitization and saved as tiff image format. Obviously, the chromaticity of these images lacked accurate correction.

Thanks to advancements in technology, such as high-resolution imaging (Srisa-ard, 2006; Fischer and Kakoulli, 2013), hyperspectral imaging (Berns et al., 2006; Elias and Cotte, 2008; Colantonio et al., 2018; Berns, 2019), and 3D imaging (Yastikli, 2007; Mączkowski et al., 2011; Simon Chane et al., 2013; Axer et al., 2016; Sitnik et al., 2016), conservators currently apply digital techniques to preserve the current state information of cultural heritage objects; they can be measured once and restored digitally without using chemicals that might irreversibly damage the objects. In addition, these techniques aim to restore lost information while performing materials analysis, color science, image processing, and so forth to explore the evolution of the fading process (Berns, 2019). The linearity of an initial image captured by digital systems makes it possible to specify the captured spectral information much more accurately than is possible with film systems. Color correction is essential in determining if a digital image is acceptable for cultural heritage applications because highly accurate images are necessary for preservation. An accurate correction of mural images is significant for conservation, preservation, restoration, and historical purposes. Early digital mural images cannot meet these requirements since they lack color management.

The discussion above highlights several issues associated with color-accurate image archives. One prime goal is to capture the colors of cultural heritage objects accurately. A high-resolution panoramic image was stitched together from small overlapping pictures captured along the customed rail tracks. Color accuracy is affected by illumination conditions, lighting uniformity, geometry conditions, white balance, and camera settings. Specifically, the following issues are addressed:

(a) The color appearance of the object shifts depending on the lighting conditions present when the image is captured and the object’s intrinsic properties, i.e., color is an unstable visual feature (Hoeben Mannaert et al., 2017).
(b) The imaging system setup is inadequate because of limited space or non-optimum lighting conditions, which can cause spatial non-uniformity in the images.
(c) The lens vignetting or light falloff with spherical aberration could result in an image that is brighter in the middle and darker around the edges because the stronger the refracted light, the more significantly the imaging signal will be reduced; this effect is especially prominent for wide-angle lenses. In most cases, the illumination is brightest in the middle of the image, with a steady decrease toward the edges (Verhoeven, 2016).
(d) Most color constancy algorithms assume that the incident illumination remains constant across a scene, but this assumption is very often not valid for real images (Cardei et al., 2002). Illumination is rarely constant in intensity or color throughout a scene.
(e) White balancing is widely applied to avoid color distortion caused by illumination changes. In general, estimates of illumination chromaticity are from space-average chromaticity or the brightest patches across scenes (Foster, 2011; Akazawa et al., 2021); it can be used for normal color calibration purposes but is insufficient for high-accuracy non-linear color correction, resulting in a lack of consistency among lighting conditions.

To address these issues and concerns, the specific objective of this study is to develop an image correction solution that employs a computational model of the human visual system to make the color appear more realistic. We specifically focus on chromatic transfer for color cast images using the color constancy theory proposed in this study. To solve the illumination non-uniformity problem, we compensated the non-uniform illumination in the scene using homomorphic filtering to eliminate illumination variations. The superior performance of this proposed method is evaluated and compared with existing methods in cave 465 of the Mogao Grottoes. The key contribution of this work is the solution it provides to “adaptively correct” color cast images with widely varying lighting conditions, and it improves color consistency efficaciously. It is hoped that this research will contribute to a deeper understanding of cultural heritage digitization.

## Materials and methods

### Methodology overview

**FIGURE 1:**
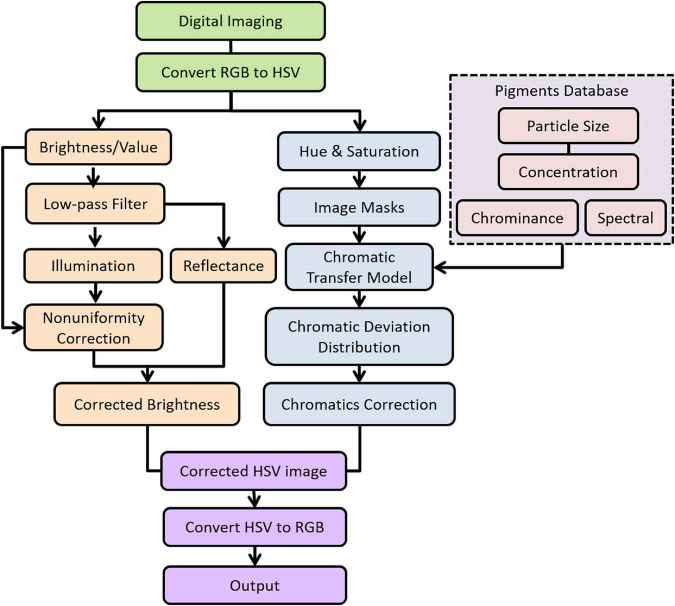
Schematic diagram for proposed methodology.

The methodology for producing a color correction of color cast image is diagrammed in Figure 1 using a mural as an example; this mural is on the south wall of cave 465 in the Mogao Grottoes. The required equipment includes an image acquisition system, photography rail track, pigment identification capabilities, and an integrating sphere spectrophotometer. Two paths radiate from the murals.

Path 1 began with the mural image acquisition system and included image denoising, image enhancement, geometric distortion correction, and image stitching. Next, the image was converted to hue, saturation, and value (HSV) color space from red, green, and blue (RGB) space, which was defined for the international commission on illumination (CIE) 1931 standard observer and standard illuminant D65 to resemble how humans tend to perceive color. Then, the non-uniform illumination was compensated using low-pass filtering in the brightness (value) channel (Section “Compensation algorithm for non-uniformity illumination”). It should be noted that the separability of the scene’s illumination and reflection components were processed in the image frequency domain.

In Path 2, the palette was defined using analytical techniques and documentation by conservation scientists. Samples with different particle sizes and concentrations were prepared for the palette following the essential techniques of Chinese mural painting, and the chromaticity and spectral properties were measured to identify the pigments (Section “Optical database”). Image masks were made for pigment segments of murals that were corrected. The HSV color coordinates were transformed into a pigment map mask using chromaticity characteristics. The size of the image mask is a compromise between minimizing the range of colors and maximizing the accuracy of the pigment map (Section “Color cast correction based on color constancy”). Moreover, the selected pigments must result in a colorimetric match for the specific illuminant and observer.

An assumption was that the concentration of the colorant was constant throughout the masks. The change in the hue component channel of the HSV color space between the mask area and the reference pigment was calculated next. These differences were converted to CIEDE2000 color differences. Pigments were selected from the optical database resulting in the closest chromaticity match for the specific illuminant and observer. The color cast correction model was constructed to complete the color compensation of relevant pigment colors. Finally, all the colors in the mask area were translated into realistic colors.

### System setup

The Mogao Grottoes is a world cultural heritage site located in northwest China that is famous for the exquisite murals and Buddhist sculptures kept inside the caves. The Mogao Grottoes have 735 caves, more than 45,000 square meters of murals, and 2,415 colored sculptures of different sizes. They provide abundant vivid materials depicting various aspects of medieval life, such as farming, textiles, war, architecture, marriage, funerals, daily dress, arts, and commercial activities in ancient China.

**FIGURE 2:**
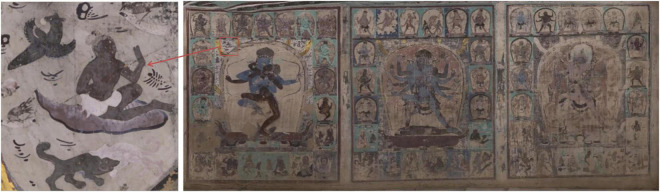
The mural image of the mandala from Cave 465, Mogao Grottoes (Yuan dynasty, 1271–1368 AD).

The approach in this paper is used to correct the mural color of the three-paved double-body mandala on the south wall of Cave 465, Mogao Grottoes, Dunhuang, Yuan Dynasty [1271–1368 anno domini (AD)], which is the earliest and most complete Tibetan-style mural outside of the Tibetan area, with a size of 13.13 m by 5.3 m, as shown in Figure 2. In the double mandala image on the east side, the male body is blue with three eyes and four arms, and the other three faces are green, gray-brown, and black; the female body is a black-brown Buddha Dakini with four hands on one face. In the double mandala in the middle, the male body is reddish-brown with three faces and six arms, the other two faces are yellow-brown and light blue, and the female body is blue with one face and six arms. In the double body mandala on the west, the male body has three faces and six arms, the body color is cyan, the rest of the face is dark brown and pink, and the female body is pink. Each mandala portrait is surrounded by small panels of deities and Great Adepts.

The developed image acquisition system included a commercial trichromatic camera (Canon EOS 1D X Mark III) with 14-bit digitization and 21 MP pixels. The lens was a Canon EF 50 mm USM. Two Elinchrom Ranger Free Lites mounted with translucent diffuse reflectors were arranged at an angle of approximately 45° to the capturing area of the murals. The imaging plane of the digital camera was set to be approximately parallel to the sample placement plane. Ten percent of the illumination deviation falls within the mean distribution. The camera was mounted on a rail track slider to capture images horizontally; each image corresponded to 0.15 square meters. After white balance, color management, and geometric distortion correction, 578 overlapped adjacent pictures were digitally stitched into a large image with a resolution of 150 dpi.

### Optical database

A database of chromaticity and spectral reflectance for the mural palette began with the image cast correction of Cave 465, Mogao Grottoes. The palette was approximated using several color pigments (Su et al., 1996; Wu, 2003; Kogou et al., 2020). The red pigments were cinnabar, red ochre, and red earth (iron oxide red). The orange pigment was Vermilion, which had a beautiful and intense tint, but it is chemically unstable and contains lead, resulting in the color fading to brown or black with the passage of time. Yellow pigments were mainly massicotite, laterite, and orpiment, in which massicotite has poor lightfastness and soon faded from yellow to coffee. The green pigments were mineral green and chlorocopperite, which have stable chemical and optical properties. The blue pigments were indigo and azurite. The white pigments were chalk and gypsum. The black pigments were discolored carbon and PbO_2_.

To adaptively and accurately correct the mural, some reference pigments commonly used in the mural are essential. The reference pigment selection is significant in at least two major aspects of consideration, chemical and chromaticity properties.

Of the many colorants in the mural, red earth, lazurite, mineral blue, mineral green, limestone, and calcite, are all capable of maintaining the same color in chroma with time. However, ochre and ultramarine are faded or darkened; some pigments, such as miniumite, lithargyrum, massicot, and white lead, have poor chemical stability; hence, their color would change under ultraviolet light, high temperatures, and humidity. These changes have dramatically influenced the mural’s color appearance, because these fading pigments cause the colors in the faces and skin to change from pink to brown. Some palette pigments are summarized in Table 1.

**TABLE 1:** The structure and properties of pigments in the Mogao Grottoes.

No.	Pigment name	Original color	Chemical structures	Current color	Result
1	Gypsum	White	CaSO_4_.H_2_O	White	Invariant
2	Chalk	White	CaCO_3_	White	Invariant
3	Orpiment	Yellow	AsS_3_ → AsO_3_	Light yellow	Faded
4	Ochre	Brownish red	Fe_2_O_3_ → Fe_3_O_4_	Dark red	Faded
5	Ultramarine	Blue	Na_6_Al_4_Si_6_S_4_O_20_	Light blue	Faded
6	Mineral green	Light green	CuCO_3_⋅Cu(OH)_2_	Green	Invariant
7	litharge	Yellow	PbO → PbO_2_	Coffee	Changed
8	Cinnabar	Red	HgS (Hexagonal system) → Isometric system	Black	Changed
9	Lead white	White	Pb_3_(OH)_4_CO_3_ → PbO_2_	Coffee	Changed
10	Vermilion	Orange	Pb_3_O_4_ → PbO_2_	Black	Changed

Dry pigments, such as vermilion, orpiment, litharge, cinnabar, azurite, ultramarine, and mineral green were dispersed in animal-derived gelatin-producing paints. Some pigments have many particle sizes, such as the minerals green, mineral blue, ultramarine, and cinnabar. Each pigment was mixed with titanium dioxide to achieve a different concentration of tints and applied to a smooth white substrate at a thickness resulting in opacity. For each pigment, there were at least ten mixture patches with white paint at weight concentrations of 0, 10, 20, 30, 40, 50, 60, 70, 80, 90, and 100%. Additionally, tint ladders were created following the essential techniques of Chinese mural painting.

The spectral reflectance and chromaticity of all the patches were measured by an X-Rite Ci64 UV portable integrating sphere spectrophotometer from 400 to 700 nm at intervals of 10 nm over the wavelength range. Measuring the specular component included (SCI) would capture accurate color data from the sample and negate the effect of surface appearance to measure only color. It makes little or no difference if the patches are mirror-like or matte in appearance. The measurement aperture of the spectrophotometer was 3.5 mm in diameter, and colors within the aperture were averaged spatially.

**FIGURE 3:**
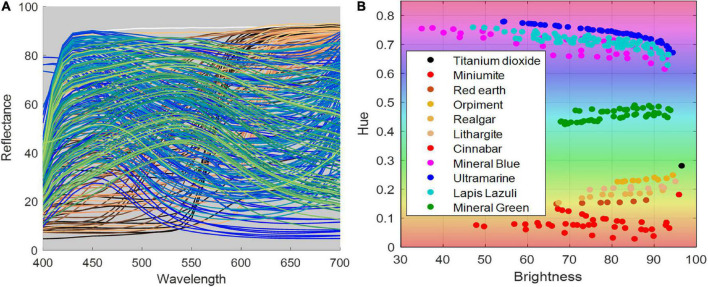
The spectral and chromaticity properties of the pigments. **(A)** The spectral reflectance, **(B)** the chromaticity coordinates in the brightness-hue plane in HSV color space.

**FIGURE 4:**
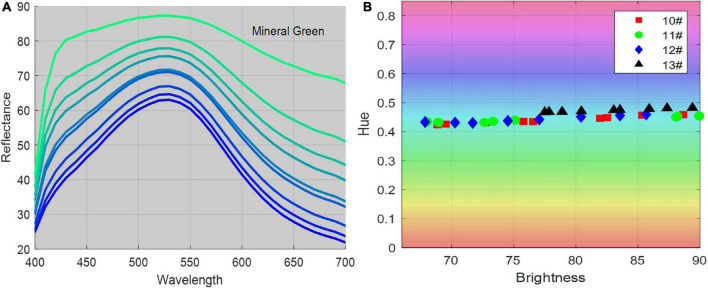
Tint ladders of the mineral green pigment. **(A)** The spectral reflectance, **(B)** the chromaticity coordinates in the brightness-hue plane in HSV color space.

Figure 3 illustrates the spectral reflectance and color distribution of patches plotted in the HSV color space. In the right color distribution subgraph, the horizontal axis presents the brightness component in HSV space, and the vertical axis presents the hue component, that is, the color type specified by the dominant wavelength of the color, such as red, yellow, green, and blue. It has been shown that some pigments overlap in hue components in high brightness, such as red earth, cinnabar, ultramarine, and vermilion. Blue pigments, such as mineral blue, have varied chromaticity at different granularities and concentrations. In contrast, only green pigments, such as mineral green, have a stable hue at different concentrations and partial sizes (10#, 11#, 12#, 13#), ranging from 0.4 to 0.5 overall pigments, as shown in Figure 4, and were suitable for image segmentation and color correction based on color constancy.

### Compensation algorithm for non-uniformity illumination

In terms of chromaticity, an image observed by people is a visual perception of color stimuli formed by light irradiated on the object’s surface; the light is typically absorbed predominantly at some wavelengths and reflects or transmits light at other wavelengths; it is well-known as the illumination-reflectance model. That is, images captured in complex scenes can be highly degraded due to unstable lighting conditions. Illumination distributions are typically very slow across an image compared to reflectance, which can change quite abruptly at object edges (Gonzalez and Woods, 2018); this difference is the key to separating the illumination component from the reflectance component.

The HSV (Hue, Saturation, and Value) color model is normally used because of its similarities to how humans tend to perceive color; the brightness is roughly analogous to the stimulus after illumination irradiation and object reflection. We, therefore, corrected colors by estimating the scene’s overall illumination in the HSV brightness channel.

The first step in this process is to convert the RGB image into HSV color space, and the brightness channel is used to separate image luminance from color information. In general, the light stimulus can be regarded as the product of the illumination of the scene and the reflectance of the objects; i.e.,


(1) I⁢(x,y)=L⁢(x,y)⁢R⁢(x,y)

where I(x,y) is the light stimulus at each point (x,y), L(x,y) is the scene illumination resulting from the lighting conditions at the time of image capture, and R(x,y) is the reflectance arising from the properties of the scene objects themselves. In homomorphic filtering, we first transform the multiplicative components into additive components by moving to the *log* domain.


(2) ln⁡(I⁢(x,y))=ln⁡(L⁢(x,y))+ln⁡(R⁢(x,y))

The Fourier transform is then used on both sides of the upper expression;


(3) Γ⁢(ln⁢(I⁢(x,y)))=Γ⁢(ln⁢(L⁢(x,y)))+Γ⁢(ln⁢(R⁢(x,y)))

or


(4) $$\text{F}\,\left(u,v\right)={\text{F}}_{\text{L}}\,\left(u,v\right)+{\text{F}}_{\text{R}}\,\left(u,v\right)$$

where F_L_(u,v) and F_R_(u,v) are the Fourier transforms of ln(I(x,y)) and ln(R(x,y)), respectively. F(u,v) is the Fourier transform of the image being filtered.

As previously stated, with the low frequencies of the Fourier transform of an image with illumination and the high frequencies with reflectance, a separation can be gained over the illumination and reflectance components with a homomorphic filter. This control requires the specification of a low-pass filter H(u,v) in the frequency domain to extract the low-frequency illumination component while preserving the high-frequency reflectance component. Then, we process F(u,v) using a Gaussian low-pass filter function H(u,v) from

(5) $$\text{G}\,\left(u,v\right)=\text{H}\,\left(u,v\right)\,\text{F}\,\left(u,v\right)=\text{H}\,\left(u,v\right)\,{\text{F}}_{\text{L}}\,\left(u,v\right)+\text{H}\,\left(u,v\right)\,{\text{F}}_{\text{R}}\,\left(u,v\right)$$

where G(u,v) is the Fourier transform from the image that has been processed. The H(u,v) filter used in this procedure is the Gaussian low-pass filter defined as


(6) $$\text{H}\,\left(u,v\right)={\text{e}}^{-\frac{{\text{D}}^{2}\,\left(u,v\right)}{2\,{\text{D}}_{0}^{2}}}$$

where D(u,v) is the distance from the origin of the center transform at point (u,v) in the frequency domain and *D*_0_ is the cutoff distance measured from the origin, which determines the bandwidth of the low-frequency band that will be filtered out. To obtain the actual results, the following formula must be returned to the spatial domain:


(7) $$\text{G}\,\left(x,y\right)=\Gamma^{-1}\,\left(\text{G}\,\left(u,v\right)\right)=\Gamma^{-1}\,\left(\text{H}\,\left(u,v\right)\,{\text{F}}_{\text{L}}\,\left(u,v\right)\right)+\Gamma^{-1}\,\left(\text{H}\,\left(u,v\right)\,{\text{F}}_{\text{R}}\,\left(u,v\right)\right)$$

The next step is to apply an exponential function to invert the log-transform at the beginning of the process and obtain the homomorphic filtered image g(x,y), which is denoted by


(8) $$\text{g}\,\left(x,y\right)={\text{e}}^{\text{G}\,\left(x,y\right)}$$

Finally, the illumination compensation model can be expressed as follows.


(9) $$\dot{\text{I}}=\frac{1}{\text{MN}}\,\sum_{\text{x}=1}^{\text{M}}\sum_{\text{y}=1}^{\text{N}}\text{I}\,\left(x,y\right)$$

(10) $${\text{I}}_{\text{output}}\,\left(x,y\right)=\frac{\text{I}\,\left(x,y\right)*\dot{\text{I}}}{\text{g}\,\left(x,y\right)}$$

where I(x,y) is the brightness image in the HSV color space, I_output_(x,y) is the corrected brightness image, and İ is the mean value of the brightness channel.

Finally, the processed image is converted back into the RGB color space.

### Color cast correction based on color constancy

The human visual system exhibits some color constancy that can keep the color perception of a scene constant when the illumination changes. Since cameras do not intrinsically have this ability, white balancing is widely applied to avoid color distortion caused by illumination changes. However, white balancing is not enough to achieve a more accurate and professional color correction, resulting in a lack of consistency among lighting conditions. Color constancy, therefore, is a feasible approach to correcting the mural’s color in a way that uses pigments with stable optical properties in chromaticity. As indicated previously, the HSV color space does a substantially better job mimicking how humans interpret color than the standard RGB color space. Hue is the primary chromaticity property that allows distinguishing pigments of different colors. To address these color casts, the following steps are taken:

The first step in this process is to convert an input image from the RGB color space into the HSV color space.

A pixel-wise mask is then created based on the angular character in the hue coordinate system dividing an image into its constituent parts or pigment colors. That is, image masks are made of areas identified for hue-based color cast correction.

In the follow-up phase, the mean and standard deviation (SD) of hue channels are computed for the cast color and reference color. For a mask image of pigments, formal definition of mean and SD are given by Eqs 11, 12:


(11) $${\dot{\text{H}}}_{\text{mask}}=\frac{1}{\text{MN}}\,\sum_{\text{y}=1}^{\text{N}}\sum_{\text{x}=1}^{\text{M}}{\text{H}}_{\text{mask}}\,\left(x,y\right)$$

(12) $$\sigma =\sqrt{\frac{1}{\mathrm{MN}-1}\,\sum_{\text{y}=1}^{\text{N}}\sum_{\text{x}=1}^{\text{M}}{\left|{\text{H}}_{\text{mask}}\,\left(x,y\right)-{\dot{\text{H}}}_{\text{mask}}\right|}^{2}}$$

where H_mask_(x,y) represents the pixel value in the hue channel of the color cast image, (x,y) denotes the coordinates of the image, Ḣ_mask_ represents the mean value of hue channels, σ represents the SD. M,N presents the size of the pixels in mask, respectively. Then, we scale the hue channel by the ratio determined by the respective SDs and add in the mean of the hue channel to obtain an image of the cast. The color correction model is defined as


(13) $${\text{H}}_{\text{corr}}\,\left(x,y\right)=\frac{\left({\text{H}}_{\text{mask}}\,{\left(x,y\right)}_{-}\,{\dot{\text{H}}}_{\text{mask}}\right)\,\sigma_{\text{patches}}}{\sigma_{\text{mask}}}+{\dot{\text{H}}}_{\text{patches}}$$

where H_corr_(x,y) represents the corrected value in the Hue channel, Ḣ_patches_ represents the mean hue value of reference patches, σ_mask_ and σ_patches_ represent the SD of the target image and reference patches, respectively, and the reference patches are of the mineral green pigment in the pigments database. The illuminant chromaticity change is given by:


(14) $$△\,\text{H}\,\left(x,y\right)={\text{H}}_{\text{corr}}\,\left(x,y\right)-{\text{H}}_{\text{mask}}\,\left(x,y\right)$$

After that, in order to estimate the scene’s illuminant chromaticity distribution, a two-order polynomial surface fitting model of illumination chromaticity change △H(x,y) is performed based on the mask defines the list of pixels.


(15) $${\text{H}}_{\text{poly}}\,\left(x,y\right)={\text{a}}_{1}+{\text{a}}_{2}\,\text{x}+{\text{a}}_{3}\,\text{y}+{\text{a}}_{4}\,{\text{x}}^{2}+{\text{a}}_{5}\,{\text{y}}^{2}+{\text{a}}_{6}\,\text{xy}$$

where, (x, y) represents the pixel position of the image, respectively, H_poly_(x,y) represents the scene’s illuminant chromaticity distribution, a_1_, a_2_, …a_6_ represents the surface fitting parameters. In particular, the chromatic components are changed by moving their deviation distributions caused by illumination changes. The corrected image H_new_(x,y) is obtained from the original image H(x,y) by:


(16) $${\text{H}}_{\text{new}}\,\left(x,y\right)=\text{H}\,\left(x,y\right)-{\text{H}}_{\text{poly}}\,\left(x,y\right)+{\dot{\text{H}}}_{\text{poly}}$$

where, Ḣ_poly_ is the mean value of the polynomial image.

Finally, the channels are merged back together and converted back into the RGB color space from the HSV space.

## Experiment and results

In this section, the proposed method is implemented and compared with the currently existing methods. We conducted experiments to confirm the effectiveness of the proposed method.

### Evaluation of reducing lighting effects

**FIGURE 5:**
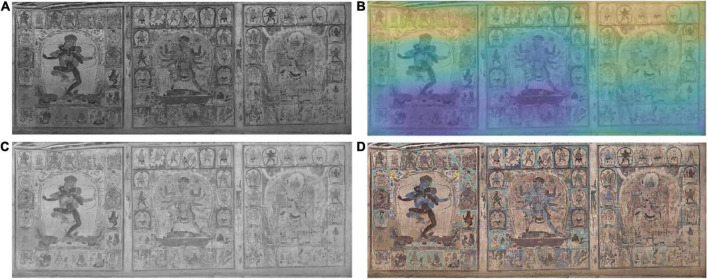
The intermediate results of illumination compensation for murals taken under ambient lighting conditions. **(A)** The brightness channel in HSV space, **(B)** estimated illumination field, **(C)** illumination compensation in brightness channel, **(D)** restored mural image by illumination compensation.

In this experiment, the effectiveness of illumination compensation was demonstrated by using homomorphic filters. Figure 2 shows the main reason for developing this technique. The original digital mural image shows obvious severe vignetting and darkening distortion on the bottom side, and in the center of the middle portrait, the illumination changes gradually from the bottom to the top. Figure 5 illustrates the intermediate results of illumination compensation for murals taken under ambient lighting conditions, where the estimated illumination field presents obvious dark in left-bottom while light in upside (see Figure 5B). The result demonstrates that the corrected image is a very nice uniform image of the same scene with the same lighting (Figure 5C). By comparing the original and the compensated images, we can see that the gradual change of illumination in the original image has been corrected to a large extent on the bottom.

**FIGURE 6:**
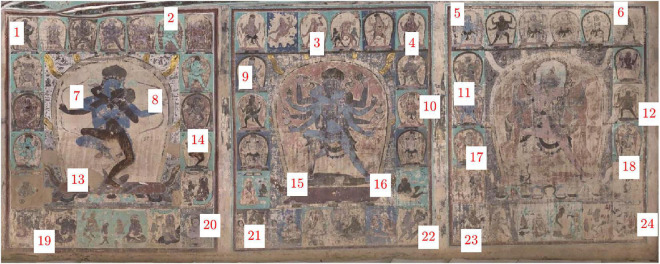
Coordinates of selected reference samples in the basal layers of the murals.

To verify the proposed approach, the performance of non-uniform compensation with a different method [including the Gaussian process (Kier, 2009) and a morphology method (Gonzalez and Woods, 2018)] was evaluated in terms of statistics. The color accuracy was dated at 24 color regions in the basal layers across the murals, as illustrated in Figure 6. All results are given in Table 2: comparing the evaluation results of different methods, it was observed that our proposed method achieves the best performance, i.e., the smallest range between maximum and minimum and the lowest SD. In more detail, the illuminance distribution across the mural image after compensation was in the range of 0.106, and the lowest SD was 0.031, which means that the compensated illuminances are all concentrated around the mean. Moreover, a very nice uniform image of the same scene is obtained. It has the largest entropy value of 6.747, where entropy is used to measure the amount of information within an image. High entropy values indicate greater randomness while low entropy values result from a more uniform image in the brightness channel. The entropy equation is as follows.


(17) Range=Maximum-Minimum

$$\text{Entropy}=-\sum_{\text{i}=1}^{\text{n}}{\text{p}}_{\text{i}}\,\log_{2}\left({\text{p}}_{\text{i}}\right)$$

**TABLE 2:** Evaluation of reducing the lighting effects.

Method	Mean	Minimum	Maximum	Range	SD	Entropy
Original	0.559	0.463	0.647	0.184	0.055	6.666
Our method	0.652	0.593	0.699	0.106	0.031	6.747
Gaussian	0.534	0.471	0.612	0.141	0.037	7.050
Morphology	0.647	0.467	0.769	0.302	0.087	7.110

where i denotes brightness level and p_i_ denotes the probability associated with brightness level. It can be concluded that the proposed method produces better illumination uniformity and contrast in the enhanced images.

### Evaluation of color cast correction based on color constancy

Another common problem raised in the previous section concerns removing color casts based on color constancy theory. It was feasible to correct the mural’s color in a way that uses pigments with stable optical chromaticity properties. Since the hue channel in HSV space is an intrinsic property of surfaces and remains approximately constant under variations in illumination, this should make image segmentation easier on the basis of quantities. A pixel-wise mask was then created based on the hue in HSV space, and the image was divided into its constituent parts or pigment colors.

**FIGURE 7:**
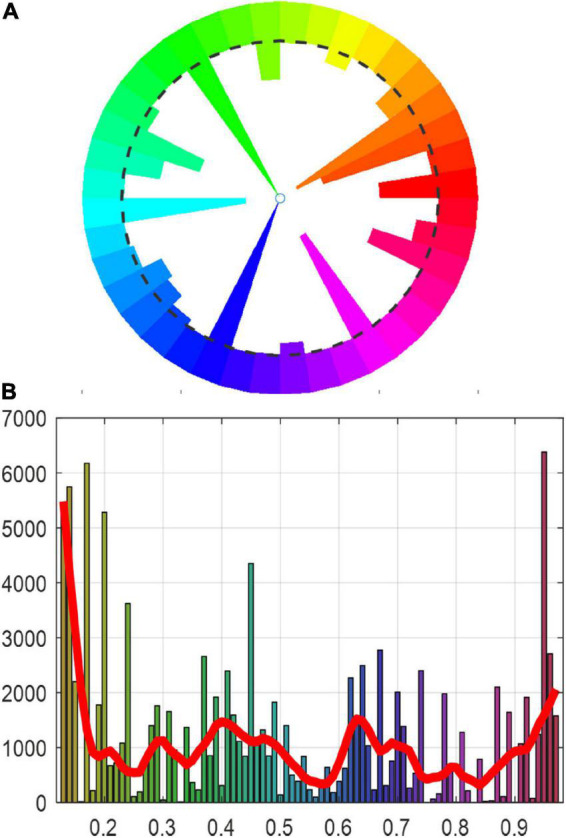
Hue histogram of Cave 465, Mogao Grottoes. **(A)** Hue polar histogram, **(B)** LOWESS plot of the hue histogram.

**FIGURE 8:**
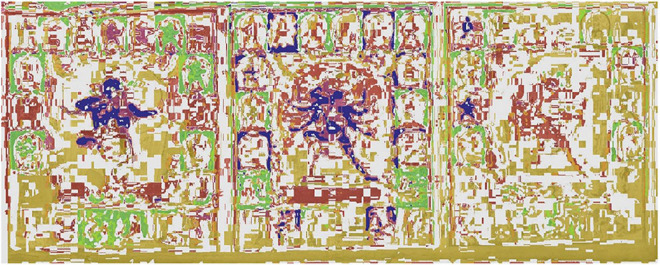
Image masks; the masks for each area are shown in the figure as solid colors.

Figure 7 illustrates the statistical diagram of the hue distribution of the mural image. The *x*-axis represents hue values and the *y*-axis represents the number of pixels. The histogram follows a multimodal distribution, and each peak represents the most common color of the pigments. The hue of the mural image is mainly composed of red, orange, cyan, blue, and purple. The mural image was then segmented into several different regions, each corresponding to one of the peaks. Despite considerable variations in brightness between the mineral green pigments in the original image, the result gave rise to one major region, as shown in Figure 8.

The color cast correction based on color constancy requires that the reference pigment has color stability chromatic properties; i.e., the chromaticity value of the pigment does not change significantly due to the granularity and concentration. As indicated previously, the mineral green pigment meets the requirements (Su et al., 1996).

**FIGURE 9:**
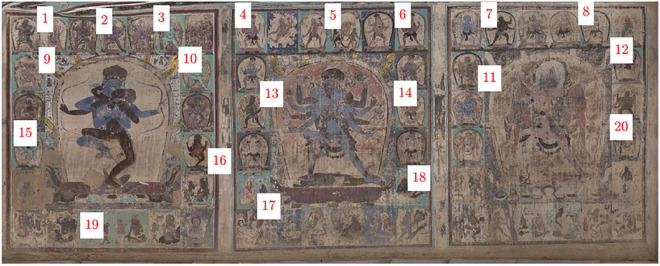
Coordinates of selected reference samples in the mineral green areas.

To verify the proposed method, 20 color regions containing mineral green were chosen for the evaluation of color correction, all areas were located in the surrounding images of the portrait in Cave 465, Mogao Grottoes, as shown in Figure 9.

**FIGURE 10:**
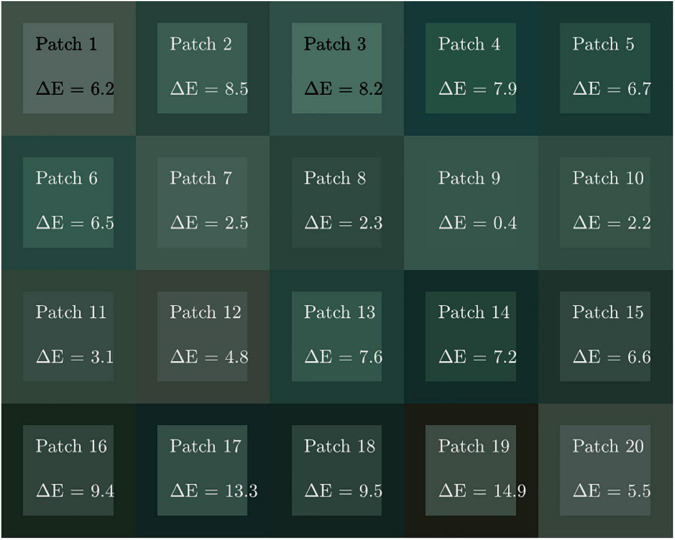
Color patch diagram of the original and corrected colors.

The results section consists of tables and figures. Figure 10 illustrates the color appearance before and after correction in the mean CIEDE2000 color difference value superimposed on each patch. The corrected color values are displayed as squares surrounded by the corresponding original color. The results revealed that the color consistency of the mineral green areas was significantly improved, particularly for some patches appeared reddish were translated into green after correction, such as patches 1, 12, and 19.

**FIGURE 11:**
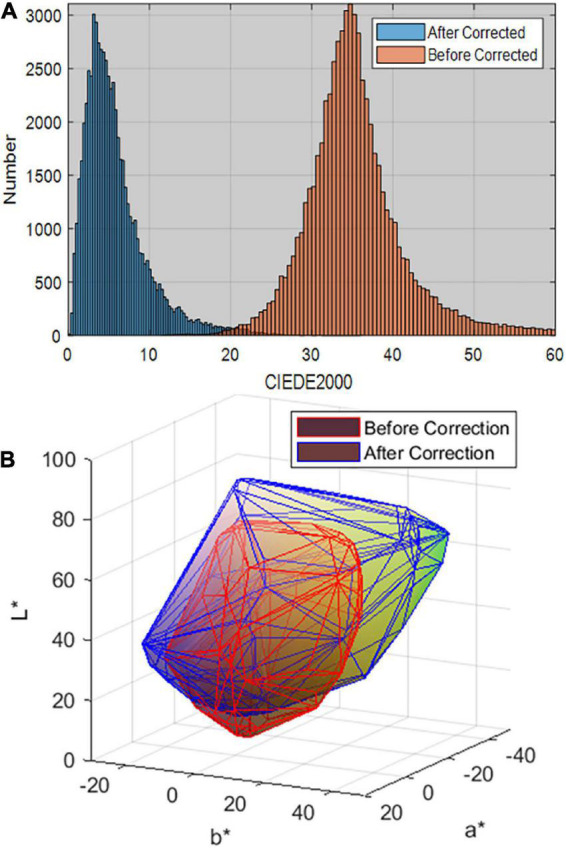
**(A)** Comparison of the CIEDE2000 color difference histogram before and after correction, **(B)** visual of the color gamut in CIE LAB color space.

The proposed method was implemented and compared with currently existing methods. These include the principal component analysis (PCA) method (Cheng et al., 2014), max-RGB algorithm (Ebner, 2007), gray world algorithm (Ebner, 2003), and traditional chromatic adaptation algorithm (Lam, 1985). The data in Table 3 demonstrate the evaluation result of the CIEDE2000 color difference. They demonstrate that our proposed method, the color correction method based on color constancy, improves the precision of color cast removal, while the traditional methods exhibit significant discrepancies without considering the intrinsic properties of some pigments. In particular, our proposed method has the smallest CIEDE2000 color difference (a mean value of 0.56, a maximum value of 1.53, a SD of 0.42) compared to the other methods, which indicates that the color variance tends to be close to the mean value and that the model has strong performance after correction. The chrominance of mineral green was significantly corrected to a reasonable chromaticity range. Figure 11 presents a CIEDE2000 color difference histogram and color gamut comparison before and after color correction for masked areas of mineral green. More generally, the original image of the scene under an incandescent lamp may lend a reddish or yellowish cast. After correction, some reddish and yellowish would be translated into green; that is, the gamut of green areas is expanded while the whole gamut is reduced. The results demonstrate that the proposed method could achieve improved consistency in the mean CIEDE2000 color difference compared with before correction.

**TABLE 3:** Comparison of the color cast correction accuracy in terms of CIEDE2000 calculated with the reference mineral green pigments.

Method	Mean	Max	Min	Rang	SD	Kurtosis	CoV
Original	1.70	4.47	0.45	4.01	1.21	2.33	0.71
Gray world	12.42	18.23	7.83	10.40	2.51	2.91	0.20
max-RGB	4.65	10.41	0.92	9.48	2.38	2.91	0.51
Chromadapt	12.96	18.23	8.47	9.76	2.44	2.68	0.19
PCA	9.91	15.87	4.77	11.10	2.70	2.76	0.27
Our method	0.56	1.53	0.001	1.53	0.42	2.66	0.75

**FIGURE 12:**
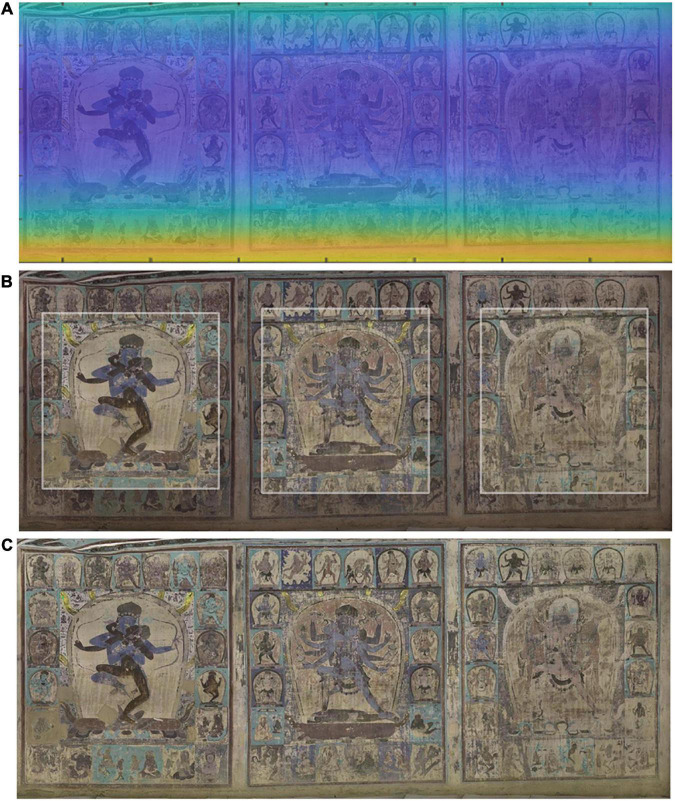
The intermediate results for color cast image correction. **(A)** The scene’s illuminant chromaticity distribution estimation, **(B)** comparison of the murals before and after color correction. The inside area of the image is color corrected area while the outside frame of the image is the image in its current condition, **(C)** the image appearance after color correction.

Building on the color correction work applied to the mineral green color, the color of the light that illuminated a scene over the whole image was constructed, and the further correction was then achieved to calibrate the chromatic value of the entire image. As illustrated in Figure 12, it is apparent that the wide color appearance between before and after color correction. The inside area shows the image appearance after color correction, while the outside frame area shows the current condition of the mural (Figure 12B). This reveals that the image’s overall color was reddish and week-lighted before correction, especially at the bottom of the murals, which was mainly influenced by the color temperature of the light source and the white balancing process during image acquisition. After correction, the overall color appearance of the corrected mural image was reasonable and acceptable, and color casts were obviously eliminated. The intelligibility of the image has been greatly enhanced, and the image is more suitable for human visual perception and contains more realistic scene colors.

**FIGURE 13:**
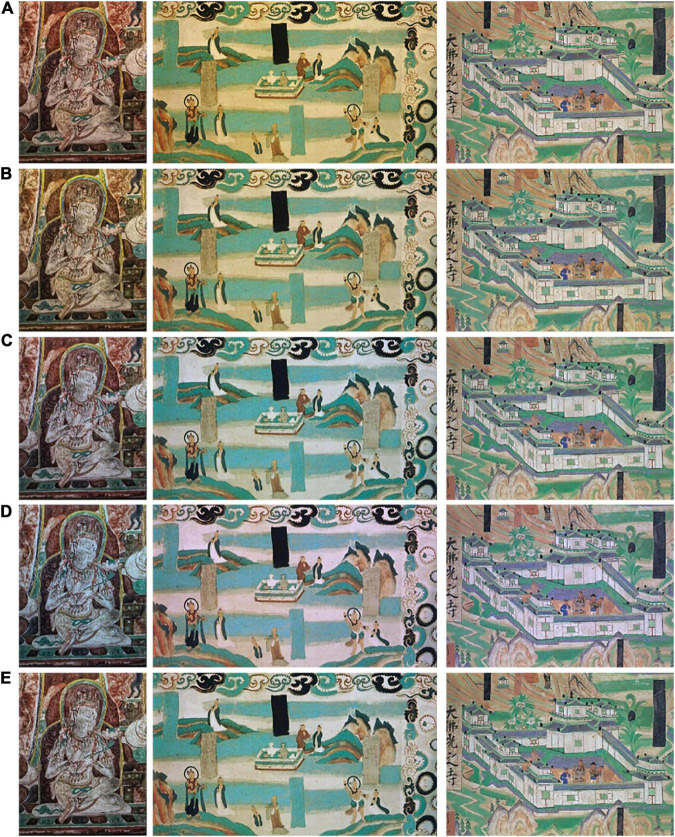
Comparison of the resulting images. **(A)** Input image, **(B)** our method, **(C)** max-RGB, **(D)** gray world, **(E)** PCA.

To compare the performance of the color correction method. The above algorithm was tested on several mural images. Figure 13 shows the calculated candidate images for the input image; when performing the visual assessment of color accuracy, the proposed method produced better tint consistency for color cast images. In particular, the pigment in the white area of murals was actually not white but faded yellowish, and the reddish or yellowish area was effectively dealt with after correction.

## Results and discussion

Restoring the correct or realistic color of a cultural heritage object is a crucial problem for imaging techniques. Currently, most methods fail to consider the intrinsic properties of pigments. In this study, we propose a new method to improve the color correction accuracy for early mural digital images with color casts. The results illustrate that the tint consistency of the corrected image can be significantly improved. The illumination compensation based on homomorphic filtering could be an effective approach to move non-uniformity illumination. The color correction model based on color constancy theory can transform a cast image into a more realistic state. The factors that have contributed to the improvement in color correction accuracy are as follows.

(1) The results from the studies imply that the spectral reflectance is an intrinsic property of objects independent of illumination; this should make image segmentation easier on the basis of chromaticity value and should not affect the overall luminance level of an image.
(2) For conservators and conservation scientists of cultural heritage, assessing color accuracy in digital images is useless when lacking concern for some pigments with color stability, such as mineral green and carbon black. Our approach is to estimate the illuminate chromaticity when mineral green pigments with hue-value stability properties as a reference are available, the experimental results show that the corrected color is more realistic.
(3) Our study demonstrates the effectiveness of this method under color constancy theory and supposing non-uniform illumination of the scene. Moreover, due to its scientific evidence, the result remains speculative and is suitable for color correction implementation.
(4) The color appearance restoration of early mural images cannot be verified and, at best, is an informed approximation when first executed. We should not lose sight of the purpose of color correction—to provide an informed impression of how a mural may have looked.

## Conclusion

The main goal of the current study was to develop a color correction solution for cast images of murals in the Mogao Grottoes. This study firstly compensated the non-uniform illumination using the homomorphic filter in the brightness channel of HSV space. Then the color cast was removed from the mural image using color constancy based on some pigments with stable optical chromaticity properties. The experiment results demonstrate that the proposed method can significantly improve the consistency of the tint and the non-uniform illumination compensated in the image frequency domain; it can achieve improved consistency compared with before correction. The contribution of this study may assist in understanding how images looked when first created to extend our knowledge of cultural heritage. These results highlight the potential usefulness of image restoration and color simulation for conservators and conservation scientists of cultural heritage. Further studies need to be carried out to evaluate the color gamut of the mask area. This colorimetric correction methodology is sufficiently accurate in color correction for cast images of murals from the early years.

## Data availability statement

The original contributions presented in this study are publicly available. This data can be found here: https://zenodo.org/record/7013743#.YwYi9XbMI2w.

## Author contributions

ZL: methodology, data collection and analysis, and writing. Y-XL: compile programs. G-AG: data collection and writing – reviewing. YK: data collection. BW: data analysis. J-XL: methodology, funding acquisition, and writing – reviewing. All authors contributed to the article and approved the submitted version.
